# Predator-Prey Interactions between Nonnative Juvenile Largemouth Bass (*Micropterus salmoides*) and Local Candidate Prey Species in the Pearl River Delta: Predation Capacity, Preference and Growth Performance

**DOI:** 10.3390/life12020295

**Published:** 2022-02-16

**Authors:** Du Luo, Minghao Ye, Dingtian Yang

**Affiliations:** 1 Pearl River Fisheries Research Institute, Chinese Academy of Fishery Sciences, Guangzhou 510380, China; 2 Guangdong Key Laboratory of Ocean Remote Sensing, State Key Laboratory of Oceanography in the Tropics, South China Sea Institute of Oceanology, Chinese Academy of Sciences, Guangzhou 510301, China; 3 College of Marine Sciences, South China Agricultural University, Guangzhou 510642, China; minghaoye@hotmail.com

**Keywords:** piscivorous fish, optimal foraging, ontogenetic dietary shifts, selectivity index, specific growth rate, food conversion ratio

## Abstract

An ontogenetic dietary shift is crucial for the survival and growth of piscivorous largemouth bass (LB). However, there is much to learn about the predator-prey interaction during the switching process. We carried out a series of indoor experiments to examine the predation capacity, predation preference, and growth performance of exotic juvenile LB feeding on candidate prey species in the Pearl River Delta. The widely distributed oriental river prawn (*Macrobranchium nipponense*), barcheek goby (*Ctenogobius giurinus*), western mosquitofish (*Gambusia affinis*), silver carp (*Hypophthalmichthys molitrix*), and mud carp (*Cirrhinus molitorella*), with relatively similar total lengths, were selected as potential prey based on their availability and habitat use. Our results show that predation capacity and preference varied quantitatively and qualitatively among prey species. The number of oriental river prawns killed was significantly less than that of fish species, comparing the 1st hour with the 24th hour (*p* < 0.01). The feeding rhythm of LB varied significantly from crayfish to fish. Numerically, Jacobs’ selection index reinforced LB’s special preference for predating *G. affinis*. Although there were obvious variations in predation capacity and feed selection, no statistically significant growth differences were detected among LB groups feeding on live *M. nipponense*, *G. affinis*, *H. molitrix*, and *C. molitorella* (*p* < 0.05). These findings suggest that the successful ontogenetic dietary shift of juvenile LB may depend on the availability and vulnerability of local fish species. Further study on the reproductive phenology of potential fish prey may help to predict LB’s establishment.

## 1. Introduction

Foraging ecology is fundamental to understanding the detailed processes of individual development and community construction [1]. The ability to capture and ingest food is the key to the acquirement of nourishment from the environment that regulates the survival and growth of young individual fish. There is often an increase in feeding diversity and the forming of species-specific traits after the larval period in the diet ontogeny for piscivores freshwater fish. Fish species that predate fish earlier gain body scale advantages [2]. Some studies found that piscine predators typically maintain the capacity to predate two to three times more prey than their average daily gain [3]. Meanwhile, morphology, behavior, and prey abundance are all crucial factors affecting the predatory selection of juvenile piscivorous fishes. Prey is not simply a passive victim, but capable of affecting predation with diverse functional traits [4]. Predator-prey interactions are critical drivers favoring the establishment of nonnative aquatic animals [5].

Largemouth bass (LB), *Micropterus salmoides*, are natively distributed in North America with a broad range from southern Florida and northern Mexico to the Great Lakes in the north [6]. As dominant piscivores in lakes and ponds throughout eastern North America, LB are one of the most popular sportfish [7]. LB are native to the North America continent, and have been introduced to many other countries in Europe, Asia, and Africa, for use as a game fish or aquaculture species [8]. LB have been adopted as a non-salmonid freshwater food fish, becoming one of the main global aquaculture species with an annual production of about 0.5 million tons. China contributed more than 99.0% of the global aquaculture production, and more than half was produced in Guangdong province. Most adult fish and seed production came from the Pearl River Delta in Guangdong province [9,10].

As early as 2000, this popular fish was listed among 100 of the world’s worst invasive species by IUCN [11]. As a top predatory fish with strong adaptability to the environment, this fish can easily live in the wild. It is reported that LB could establish in non-native waters and impact local fish communities through predation [12]. Water quality, habitat types, and prey behavior are all factors affecting the predation performance of LB [12]. Individual growth is one of the most vital factors related to the survival and size structure of populations [13]. Prey availability, as an environmental condition, commonly influences the intake and expenditure of energy for fish [14]. LB were introduced into the Pearl River Delta in 1983. Luckily, there was no report for its establishment in the wild. However, there were occasional observations of reproduction in semi-natural ponds or reservoirs. Fingerlings and juveniles are widely cultivated for recreational fishing and aquaculture, and the yields are increasing. Additionally, with the improvement of fish resource protection and climate change, the risk of establishment requires urgent assessment.

The timing of the shift to piscivore is important for the survival and recruitment of fish [7]. The predator-prey interactions, mediated survival, and growth of juvenile LB, are critical for dietary adaptation and switching. Investigating the characteristics of predatory behavior may improve our ability to understand and predict the possibility of establishment [15,16]. To explore the predator-prey interactions in foraging ecology between exotic juvenile LB and local prey species, firstly, we selected five aquatic animals as candidate prey. Then, we conducted a series of indoor experiments to survey LB’s predatory behavior, and examine the predation capacity, predation preference, and growth performance of juvenile LB. Thus, we provide insight into the detailed species-specific effect of dietary shift in juvenile LB.

## 2. Materials and Methods

### 2.1. Collection and Maintenance of Predator and Prey

The predators, 200 juvenile largemouth bass (*Micropterus salmoides*) with similar total lengths (TL) of 12.0–17.0 cm and body masses (BM) of around 30.0–40.0 g, were purchased from an aquaculture farm in Guangzhou. Five species, the oriental river prawn (*Macrobranchium nipponense*) barcheek goby (*Ctenogobius giurinus*), western mosquitofish (*Gambusia affinis*), silver carp (*Hypophthalmichthys molitrix*), and mud carp (*Cirrhinus molitorella*) were used as candidate prey, according to the assemblage of local aquatic animals. All the candidate prey animals were relatively similar in the morphology of total length (TL). The origin of individuals, recorded maximum length of mature individuals, preferred habitat, and food are also displayed in Table 1, mainly according to FishBase (https://www.fishbase.se/, accessed on 11 June 2021) [17]. Oriental river prawns, barcheek goby and western mosquitofish were collected from semi-natural ponds. Silver carp and mud carp were purchased from aquaculture farms. The TL of all the prey species ranged from 3.0 cm to 5.0 cm. Before the experiment, predators and prey were temporarily kept in stable conditions with pond water and air supplied for at least ten days and two days respectively.

### 2.2. Predation Capacity Experiment

The predation capacity of juvenile largemouth bass with TL = 13.40 ± 0.81 cm and BM = 44.33 ± 6.49 g was assessed using five prey species comprising the oriental river prawn (*M. nipponense*) barcheek goby (*C. giurinus*), western mosquitofish (*G. affinis*), silver carp (*H. molitrix*), and mud carp (*C. molitorella*). One predator fish was put into each plastic tank, filled with 0.8 m^3^ pond water at a temperature of about 23.0 °C, 24 h before adding prey to better adapt to the experimental conditions. In every group, the following number of individuals was included, 20 of *M. nipponense*, 20 of *C. giurinus*, 50 of *G. affinis*, 15 of *H. molitrix,* and 20 of *C. molitorella*. The number of preys was the same for each species among groups. The body mass, full length and body depth of each predator LB were measured. For each prey species, the body mass, full length, and body depth of five individuals were measured and the total weight of prey was recorded before they were released to each tank. A cover of polyethylene sheet was used for each box to control light and prevent the prey from escaping. One hour after the prey was put into the tank, the number of killed prey was recorded. The experiment lasted for 24 h. At the end of this experiment, all the animals were counted and measured. Predation capacity (PC) was calculated as the total number (PCN) and weight (PCW) of prey consumed by one bass in 24 h. The percentage compositions of killed individuals per species in the first hour (PCN_1_) and in 24 h (PCN) were calculated (RPCN), and used as a behavioral characteristic of feeding rhythm (FR).

RPCN = PCN_1_/PCN × 100

### 2.3. Predation Preference Experiment

We assessed the predation preferences of largemouth bass for potential local food resources. Four species, *M. nipponense*, *C. giurinus*, *G. affinis*, and *C. molitorella*, were used as candidate prey in this experiment, with the same scale as used in the previous experiment of predation capacity. Based on the predation capacity and species composition, 12, 10, 15 and 6 individuals of *M. nipponense*, *C. giurinus*, *G. affinis*, and *C. molitorella*, respectively, were used as prey in each section. Obstacles were simulated with two net pits and three bricks fixed at the bottom of the tank. After a 24 h pre-adaptation, one predator fish was released into the tank. Twenty-four hours later, we ended the trial and all the experimental animals were counted and measured. The predation preference was represented by the selection index. The forage ratio of Jacobs’ selectivity index [18], which is a modified version of Ivlev’s index, was used to evaluate the predation preferences of largemouth bass. This ratio is independent of the relative abundance and directly reflects the differential mortality rates caused by predation activity. Jacobs’ index was calculated following the equation as described in [19,20]:

D_i_ = (r_i_ − p_i_)/((r_i_ + p_i_) − 2r_i_p_i_)

where r_i_ is the eaten fraction of a given prey species i, and p_i_ is the fraction of the same prey species i to the sum of prey in the whole tank.

### 2.4. Growth Performance Experiment

The trophic transference rate was tested through an indoor growth experiment with a maximum water temperature of 20.0–24.0 °C. Largemouth bass with a TL = 15.01 ± 0.92 cm and a BM = 36.59 ± 7.17 g were randomly grouped with two in each plastic tank. Four prey species, *M. nipponense*, *G. affinis*, *H. molitrix* and *C. molitorella,* were used as food resources and added into each tank, respectively. Simulated obstacles were constructed, as mentioned before in the predation preference experiment. All tanks were covered with a nylon net to avoid prey escaping. The experiment was designed to last for one month with observations twice per day. Plenty of prey was added as soon as necessary and weighed every time. At the end of the experiment, all the predators and prey were counted and measured. The growth parameters of specific growth rate (SGR), average daily gain (ADG), and feed conversion ratio (FCR) were calculated as follows:

SGR = 100(e^g^ − 1)

g = (ln W_f_ − ln W_i_)/t

ADG = (W_f_ − W_i_)/t

FCR = Feed intake (g)/weight gain (g) = Feed intake (g)/W_f_ − Wi (g)

where, e is Euler’s number, W_f_ is the final weight of fish (g), W_i_ is the initial weight of fish (g), t is the number of feeding days and ln is the natural logarithm.

Notably, the term specific growth rate (SGR) is widely used in fishery literature. However, reports show that the most used formula cannot be interpreted as a per cent change in weight per unit of time and may be somewhat misleading [13,21,22]. In order to make comparisons with previously reported research, we still examined the parameter and expressed its value as SGR_2_.

SGR_2_ = 100(ln W_f_ − ln W_i_)/t

### 2.5. Statistical Analysis

Multiple statistical significance testing of parameters was carried out among the groups of prey species using analysis of variance (ANOVA) with a Tukey honestly significant difference (HSD) test. Differences between the mean values for various prey groups were considered statistically significant at *p* < 0.05. Statistical analyses were performed using R statistical software 4.1.1 (R Core Team 2021) [23].

## 3. Results

### 3.1. Predation Capacity

As shown in Table 2, the five prey species from four taxonomic families were all potential preferred food resources for largemouth bass, separately. The juvenile bass predated as many as 22.5 ± 3.5 *G. affinis* in 24 h (PCN), significantly more than the other four prey groups (*p* < 0.05). Juvenile bass also killed as many as 12.0 ± 1.4 *C. giurinus* in 24 h, a significant difference from *H. molitrix* numerically. In weight, the predator could only consume 2.5 ± 1.0 g of *M. nipponense* in 24 h (PCW), which was significantly less than the other prey species, except from *H. molitrix*. However, largemouth bass could efficiently eat as many as 12.6 ± 1.6 g of *C. giurinus* in one day, significantly more than for *M. nipponense* and *H. molitrix*. The data showed that there were differences in rhythms of predation. All *H. molitrix* and *C. molitorella* were killed in the first hour, while the bass only killed about 50 percent (RPCN = 55.6 ± 19.2%) of *M. nipponense* in the initial hour. The number of predated oriental river prawns (PCN) was significant less than that of fish species.

### 3.2. Predation Preference

**Figure 1:**
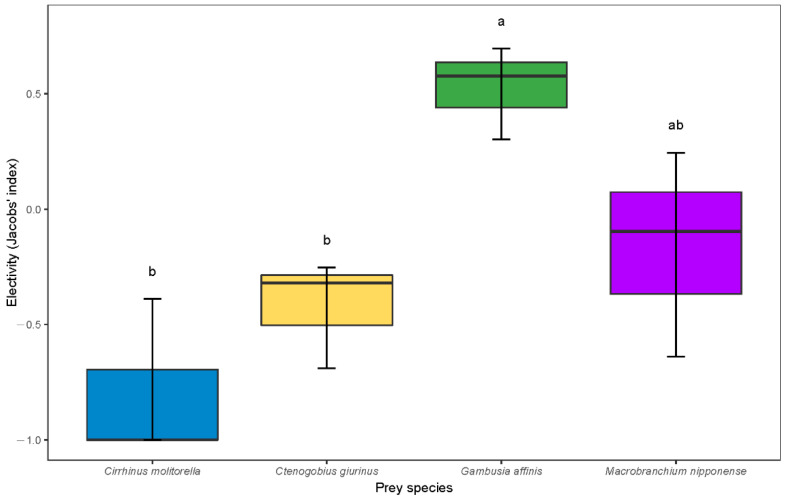
Boxplot showing predation preferences (Jacobs’ selectivity index) of juvenile largemouth bass on various prey species. Representing 50% of the central data, the box in the boxplot starts in the first quartile (25%) and ends in the third (75%), with a line inside that represents the median. Significant differences between the means are indicated by superscript letters. If two variables in the same row have the same superscript letter, the difference between the means is not statistically significant.

Our results show that all four groups of candidate species were predated by largemouth bass when they were gathered in a tank. However, there were selection effects during the predation process among the four groups, with the various selection indexes shown in Figure 1. Largemouth bass preferred *G. affinis* > *M. nipponense* > *C. giurinus* > *C. molitorella*. Numerically, largemouth bass exhibited a special preference for predating *G. affinis*. Jacobs’ index significantly differed between *C. giurinus* and *C. molitorella* (*p* < 0.05). *M. nipponense* was also well chosen by the predator, although no significant differences were detected. We observed an avoidance of predating *C. molitorella* in some tanks. There were broad variations in the standard deviation for the selectivity index.

### 3.3. Growth Performance

Largemouth bass fed with four aquatic prey species, the oriental river prawn (*M. nipponense*), western mosquitofish (*G. affinis*), silver carp (*H. molitrix*), and mud carp (*C. molitorella*), could grow well with no malformation, and behave normally. The average daily gain (ADG) of body mass was always more than 1.0 g/d (Table 3). Bass fed with *C. molitorella* gained the highest ADG, but no significant difference in ADG was detected among different prey groups (*p* < 0.05). The specific growth rates (SGR) of the groups *H. molitrix* and *C. molitorella* were relatively high compared to those of *M. nipponense* and *G. affinis*. There was no detected difference in SGR, or SGR_2_, among the four prey species. The food conversion ratio (FCR) ranged from 3.37 ± 0.65 in *M. nipponense* to a maximum of 4.33 ± 0.71 in *G. affinis*. Similarly, there were no significant differences among the four feeding resources.

## 4. Discussion

When fish have developed from fry and fingerling, in which forms they are able to feed themselves and have completed metamorphosis, to the juvenile stages, obtaining nourishment efficiently becomes extremely important in order to survive and grow [1]. Predation, in animal behavior, is a kind of ecological interaction, where the predator kills and feeds on the prey. It is a basic mechanism controlling the flow of energy between two organisms. Predator-prey interactions, as a major evolutionary driving force, are central to our understanding of adaption and community ecology [24]. The current study indicated that LB could pursue, capture and kill various aquatic animals to gain energy from the ecosystem. The introduced LB maintained their piscivorous traits as top predators. Prey availability, predation selectivity, predatory behavior, and energy transfer efficiency would be keystones for the adaptation and shift of LB into various niches [25].

The number of organisms killed by LB in 24 h (PCN) varied between different species, even though the size (TL) of prey used in this experiment was relatively similar. In addition to the quantity of individuals killed, differences in the weight of killed animals (PCW) suggested that predation capacity could be directly correlated with the prey’s body morphology. All the species included were easily captured by LB. As a top predatory fish, the body size of LB may play a major role in feeding kinematics [26]. However, we observed occasional death of LB when fed with larger sizes of *M. nipponense* in the pre-experiment. Additionally, the dropped legs of oriental river prawn under experimental conditions indicated that the shrimp might have some antipredator ability through the exoskeleton, affecting predatory efficiency.

The theory of optimal foraging behavior stipulates that natural selection will produce optimal food selection plans for animals, as well as optimal timing of looking for and switching resources. Predators are predicted to gain maximum energy in each predation [20,27]. The present research shows that the numbers of each local species killed in the first hour and those in 24 h (RPCN) differed significantly between shrimp and fish. This suggests that prey species may change their strategy of antipredator behavior with the differentiation of taxonomic categories [28]. From another perspective, variations in feeding rhythm (FR) imply that the local food resources of fish might encounter more predation risk. All of the RPCN values were above 50% and all those of fish as prey were above 80%. This was consistent with previous reports that LB predation on stocked walleye was greatest within 24 h [29]. LB was in keeping with optimal foraging theory in looking for food resources.

As a top predator, LB can easily capture prey. The scale of samples used as prey in this experiment was about one-third of LB in body length and body depth. The difference in predation preferences may be mainly due to behavioral interactions. Jacobs’ index showed that LB exhibited a special preference for *G. affinis* and *M. nipponense*. However, *C. giurinus* and *C. molitorella* were the second choices when all the candidate prey species were gathered in a tank. Crayfish are the primary food source of adult LB in Florida, but there has been a transition in dominant diet with ontogeny [15]. The introduced LB still maintains this characteristic and the presence of crayfish in local ecosystems may play a key role in nourishing alien LB. Mosquitofish could mediate the dietary transition, if the population density was high enough. However, mosquitofish were too easily captured by LB. Therefore, this species might not serve as a main diet when considering its biomass. Investigations into lake-dwelling LB in Virginia found that they ate significantly more alewives than striped bass when both species were available [30]. Other studies have also shown that bass are opportunistic feeders and tend to feed on the most frequent and abundant encounters [31]. Wild LB have been found to alternate main feeding resources as the abundance or size of prey varies [30].

Habitat usage would be another reasonable interpretation of predation preference. Cover and substrate varied among LB and those fish species with a similar ecological niche [32]. Predation pressure affected habitat choice and feeding behavior for prey fish, processes which are size-dependent for juvenile fish [33]. *C. giurinus* and *C. molitorella* were wall and bottom users, respectively. The obstacles present supplied more chances of escaping from predation, even though the coverage was not complex enough in this experiment. The effect of habitat variation is also a crucial factor in evaluating the functional traits of LB. A study from native rivers in Illinois found that bighead carp was the favorite of some piscivorous species. The native LB preferred bighead carp to silver carp, which were similar in morphology [34]. Sanft et al. [35] assumed that this scenario was partially caused by school behavior. This was consistent with yellow perch, a generalist forager with a strong predation preference for one species due to prey differences in behavior and habitat [36]. Prey personality significantly affects predator-prey interactions in species mortality and preferential selection. Predator avoidance strength might directly or indirectly depend on the predator hunting mode [37]. To describe predator-prey interactions, more detailed further studies on the personality and size-effect of local species are needed.

Feeding on the oriental river prawn (*M. nipponense*), western mosquitofish (*G. affinis*), silver carp (*H. molitrix*), and mud carp (*C. molitorella*), all LB were able to grow quickly with healthy phenotype and behavior. There were no significant statistical differences among prey species (*p* < 0.05), although the average ADG, SGR, and FCR among groups was noticeably different. This may be partially attributable to intragroup variation caused by predator personality. Data from wild-sampled brown trout reared under different conditions show that its standard metabolic rate was not positively correlated with growth in a warm regime [38]. It may partially explain why the difference in ADG did not result in a corresponding difference in SGR.

In addition, space utilization was different, even though we used similar coverage and materials in the tanks. Vegetation coverage influenced the SGR of mandarin fish, a widely distributed fish species with a similar ecological niche to LB [39]. As an aquaculture species occupying an important position, the growth of LB had been well studied when fed with compound and fresh frozen feeds. The SGR and FCR of LB fed with fresh frozen feeds were higher than for artificial compound feeds [40,41,42]. In the present study, we found that the FCR of juvenile LB fed with live bait ranged from 3.37 ± 0.65 to 4.33 ± 0.71. It was similar to that of juvenile LB fed with fresh frozen *Decapterus maruadsi,* where FCR = 4.25 [42].

LB’s diet is partitioned differently throughout its life stages [15]. Reports show that larger juveniles (≥150 mm) can survive and grow better than medium- (100 mm) or small- (55 mm) sized fingerlings when stocked in a short time. However, releasing medium-sized fingerlings is always recommended because of cost [16]. Therefore, the survival and growth of 100–150 mm juveniles would be extremely critical in adapting to the wild waters. This research demonstrates that all four local species in the Pearl River Delta could be adopted as prey of the nonnative juvenile LB during the period of ontogenetic dietary shift. Bass grow faster after switching to piscivorous behavior [7]. This indicates that theoretically these individuals would mature and further improve their predatory ability if there is enough prey in the wild waters. Under circumstances of such ecosystem complexity, a future study focusing on how predator-prey interactions are affected by prey availability, habitat use, and species-specific differences during the ontogenetic diet shift of LB would make the key processes of establishment clearer.

## Figures and Tables

**Table 1:** Taxonomic categories, origin, recorded maximum length of mature individuals (MXL, cm) and, habitat and food preference of the aquatic animal species used as prey.

No.	Species	Common Name	Family	Origin	MXL (cm)	Habitat and Food
1	*Macrobranchium nipponense*	Oriental river prawn	Palaemonidae	China	10.0	freshwater, brackish; phytoplankton, zoobenthos, detritus
2	*Ctenogobius giurinus*	Barcheek goby	Gobionellinae	China	12.1	marine, freshwater, brackish; zoobenthos, plants, zooplankton
3	*Gambusia affinis*	Western Mosquitofish	Poeciliinae	North and Central America	7.0	freshwater, brackish; zooplankton, zoobenthos, others
4	*Hypophthalmichthys molitrix*	Silver carp	Cyprinidae	China	120.0	freshwater, brackish; plants, zooplankton
5	*Cirrhinus molitorella*	Mud carp	Cyprinidae	China	55.0	freshwater; plants, detritus, zoobenthos

**Table 2:** Morphology of prey, and predation capacity of juvenile largemouth bass. Data was given in the form of mean ± standard deviation.

No.	Prey Species	TL (cm)	BM (g/ind.)	APN (ind.)	PCN (ind.)	RPCN (%)	PCW (g)
1	*Macrobranchium nipponense*	3.6 ± 0.8	0.40 ± 0.07	20	6.0 ± 3.0 ^bc^	55.6 ± 19.2 ^b^	2.5 ± 1.0 ^c^
2	*Ctenogobius giurinus*	3.7 ± 1.4	1.32 ± 0.15	20	12.0 ± 1.4 ^b^	83.2 ± 2.0 ^a^	12.6 ± 1.6 ^a^
3	*Gambusia affinis*	3.4 ± 1.2	0.29 ± 0.01	50	22.5 ± 3.5 ^a^	89.5 ± 7.8 ^a^	8.0 ± 0.1 ^ab^
4	*Hypophthalmichthys molitrix*	4.6 ± 0.4	1.70 ± 0.30	15	4.0 ± 0.0 ^c^	100.0 ± 0.0 ^a^	5.0 ± 0.3 ^bc^
5	*Cirrhinus molitorella*	3.9 ± 0.3	1.06 ± 0.09	20	8.0 ± 1.7 ^bc^	100.0 ± 0.0 ^a^	7.9 ± 2.3 ^ab^

TL: total length of prey, BM: body mass of prey, APN: the number of prey added in one tank, PCN: predation capacity (ind.) of largemouth bass as predator in 24 h, RPCN: ratio of PCN in 1 h to that in 24 h, RPCN = PCN_1_/PCN × 100, PCW: predation capacity (g) in 24 h. Different superscript letters in the same row indicate significant differences (*p* < 0.05) among groups. If variables share the same superscript letter, the difference between the means is not statistically significant.

**Table 3:** Growth performance of juvenile largemouth bass. Data was given in the form of mean ± standard deviation.

No.	Prey Species	TL (cm)	BM (g)	SGR	SGR_2_	ADG (g/d)	FCR
1	*Macrobranchium nipponense*	3.6 ± 0.5	0.40 ± 0.03	2.78 ± 0.41 ^a^	2.74 ± 0.40 ^a^	1.46 ± 0.48 ^a^	4.33 ± 0.71 ^a^
2	*Gambusia affinis*	3.4 ± 0.4	0.22 ± 0.02	2.64 ± 0.70 ^a^	2.61 ± 0.37 ^a^	1.64 ± 0.63 ^a^	3.37 ± 0.65 ^a^
3	*Hypophthalmichthys molitrix*	4.0 ± 0.2	1.24 ± 0.40	3.35 ± 0.44 ^a^	3.30 ± 0.43 ^a^	1.16 ± 0.32 ^a^	3.48 ± 1.11 ^a^
4	*Cirrhinus molitorella*	4.8 ± 0.4	1.20 ± 0.25	3.06 ± 0.65 ^a^	3.02 ± 0.63 ^a^	2.08 ± 0.57 ^a^	3.71 ± 0.15 ^a^

TL: total length of prey, BM: body mass of prey, SGR: specific growth rate in weight, SGR_2_: specific growth rate calculated using the empirical formula, ADG: average daily gain of weight, FCR: food conversion ratio. The same superscript letters (a) in the same row indicate there are no statistically significant differences (*p* < 0.05) detected among groups.

## Data Availability

The data supporting the findings of this study are available from the author upon reasonable request.
